# Association between muscle dysmorphia psychopathology and binge eating in a large at-risk cohort of men and women

**DOI:** 10.1186/s40337-022-00632-8

**Published:** 2022-07-25

**Authors:** Robin Halioua, Andrea Wyssen, Samuel Iff, Yannis Karrer, Erich Seifritz, Boris B. Quednow, Malte Christian Claussen

**Affiliations:** 1 Department of Psychiatry, Psychotherapy, and Psychosomatics, Psychiatric University Hospital Zurich, University of Zurich, Lenggstrasse 31, 8032 Zurich, Switzerland; 2 University Hospital of Child and Adolescent Psychiatry and Psychotherapy, University of Bern, Bern, Switzerland; 3 Institute of Social and Preventive Medicine, University Bern, Bern, Switzerland; 4 Experimental and Clinical Pharmacopsychology, Department of Psychiatry, Psychotherapy and Psychosomatics, Psychiatric University Hospital Zurich, University of Zurich, Zurich, Switzerland; 5 Private Clinic Wyss AG, Muenchenbuchsee, Switzerland; 6 Adult Psychiatry, Psychiatric Services Grisons, Chur, Switzerland

**Keywords:** Muscle dysmorphia, Binge eating, Muscular ideal, Eating disorder, Body image, Bulimic feature

## Abstract

**Background:**

Current research on muscle dysmorphia (MD) has focused on restrained eating behaviors and has adopted a primarily male perspective. Despite initial evidence, the role of possible binge eating associated with MD has only been scarcely investigated. To extend the transdiagnostic and cross-gender approaches and address the dearth in research related to MD, this study investigated the association between MD psychopathology and binge eating in men and women.

**Methods:**

This study investigated the association between MD psychopathology and binge eating in both men and women. Participants were a sample of 5905 men (n = 422) and women (n = 5483) social media users aged 18–72 years. They completed an online survey that included self-report measures assessing demographics, binge eating, MD psychopathology, and drive for thinness and leanness. Binge eating was assessed using the diagnostic questions of the validated German version of the Eating Disorder Examination-Questionnaire. The Muscle Dysmorphic Disorder Inventory (MDDI) was used to assess MD psychopathology. A total score of > 39 was set as a cutoff to define an “MD at-risk” state for both men and women. Hierarchical logistic regression analysis was used to analyze the association between MD psychopathology and binge eating.

**Results:**

MD psychopathology was significantly positively associated with binge eating in both men and women. Among the three MDDI subscales, only appearance intolerance was significantly associated with MD, and drive for size and functional impairment were not associated. MD at-risk status yielded a predicted probability of binge eating of 25% for men and 66.9% for women. The increased probability of binge eating associated with MD at-risk status was mainly accounted for by appearance intolerance in men and drive for thinness in women.

**Conclusion:**

MD psychopathology is positively associated with binge eating in both men and women. Binge eating episodes should therefore form part of the clinical assessment of MD.

## Plain English summary

Muscle dysmorphia is a mental disorder in which those affected are constantly preoccupied with being insufficiently muscular. Although there is initial evidence that binge eating may play a role in the clinical presentation of muscle dysmorphia (MD), this has not been investigated. In addition, MD has rarely been studied in women. We conducted a study involving 5905 men (n = 422) and women (n = 5483) using self-report questionnaires to examine the association between binge eating and symptoms of MD. We found that symptoms of MD and binge eating are positively associated. According to our model, two-thirds of women and one-quarter of men at-risk for MD exhibit binge eating. Binge eating episodes should therefore form part of the clinical assessment of MD.

## Background

In modern times, the image of an ideal body implies an increasingly lean and more muscular form for women and men [1]. While men aim for a large and muscular body with broad shoulders [2], women prefer a toned and thin figure [3]. The pursuit of a muscular physique might lead to a disorder that was first described in 1993 as muscle dysmorphia (MD) [4].

The main clinical feature of MD is the chronic preoccupation with being insufficiently muscular. In women, it refers to both being insufficiently muscular and lean [5]. People affected by MD adopt a consuming lifestyle characterized by a rigid exercise and diet regime. Initially placed on the eating disorder (ED) spectrum [4], MD is now considered a subtype of a body dysmorphic disorder within the obsessive–compulsive and related disorder spectrum in the fifth edition of the Diagnostic and Statistical Manual of Mental Disorders (DSM-5) [6]. Although Pope et al. [7] acknowledged that MD resembles ED in many ways, they argued that those affected by MD tend to achieve greater muscularity primarily through exercise and not through diet. The focus on diet was considered secondary, which was supposed to distinguish MD from an ED.

Studies criticized this nosological classification. Following a transdiagnostic perspective, Murray et al. [8] argued that the conceptualization of MD as an ED could be of clinical relevance, as it would provide the male perspective of ED pathology. Similarities with anorexia nervosa (AN) were particularly emphasized, and it was argued that AN and MD might represent the opposite poles of a continuum in which AN would represent the pathological pursuit of a thin body ideal and MD the pathological pursuit of a muscular body ideal [8, 9].

In addition to the controversial nosological classification, research on the pursuit of the muscular ideal is influenced by gendered conceptions. Consequently, MD has been studied primarily from a male perspective [10, 11]. Pathologies related to the pursuit of the muscular ideal that affects women are rarely considered in MD and are instead discussed in the context of ED.

From a transdiagnostic and cross-gender perspective, the body-modifying behaviors to achieve the muscular ideal are the same for men and women. While muscularity is primarily achieved through exercise, the level of thinness and/or leanness is often regulated by caloric intake. Studies have shown that the *drive for muscularity* and the *drive for thinness* are not mutually exclusive and co-exist in both men and women [12] and are associated with disordered eating [13, 14]. Restrained eating behaviors, such as calorie counting, are thus observed in both sexes in thinness- and muscularity-oriented disordered eating [15, 16].

Although body-changing behaviors are theoretically the same for men and women, it cannot be assumed that they apply equally to both because the characteristics of the muscular ideal differ between the two sexes. This is supported by studies showing that, on average, men exhibit a higher *drive for muscularity* [2] and a lower *drive for thinness* [17] than do women. They also differ in the composition of the Muscle Dysmorphic Disorder Inventory (MDDI), with men exhibiting a highe*r drive for size* and a lower *appearance intolerance* compared to women [18]. Therefore, the clinical presentation of MD might differ between men and women.

Despite the transdiagnostic perspective, another knowledge gap in the current research is the focus on restrained eating behaviors. The association between binge eating and MD has only been scarcely investigated [19]. Binge eating occurs in a wide range of EDs. It is a key diagnostic feature of bulimia nervosa and binge eating disorder but can also occur in AN, the binge eating/purging type [6]. Considering that the pursuit of the muscular ideal is often associated with muscularity-oriented eating, consisting of intentional, periodic overeating to build muscle mass ("bulking") [20], severe MD psychopathology might lead to high levels of overeating, loss of control, and thus binge-eating. Accordingly, prior studies indicated that binge eating is a factor in the pursuit of the muscular ideal. Competitive bodybuilders report a significantly higher incidence of binge eating compared to regular gym-goers [21]. In a case report, Murray et al. [22] described an adolescent man with both bulimic and MD-type symptomatology and suggested further investigations.

To extend the transdiagnostic and cross-gender approaches and address the dearth in research related to the pursuit of the muscular ideal, this study investigated the association between MD psychopathology and binge eating in men and women. Based on previous evidence [21, 22], we hypothesized that MD psychopathology is positively associated with binge eating in both men and women. Given the difference in MD psychopathology between men and women [18], we further hypothesized that the magnitude of the positive association differs between the sexes.

## Methods

### Setting

This research is a part of an ongoing longitudinal study examining body satisfaction and eating behaviors in the online fitness community. It is based on data from the first survey and uses a cross-sectional design.

Participants were men and women (aged ≥ 18 years) who used social media and followed fitness influencers on Instagram. They were recruited online through the distribution of a survey link via three German-speaking male fitness influencers with a total of 316,000 followers. Potential participants were informed that the study aimed to examine the relationship between body satisfaction, eating, and exercise behaviors. Recruitment lasted 14 days, beginning on May 14 and ending on May 28, 2021. After informed consent was obtained, participants completed the questionnaires anonymously, which took approximately 15 min. The questions were divided into different sections (study ID, demographics, sporting activities, and questionnaires). All questions had to be answered in a section to move to the next. Participants did not receive any compensation for participating. A REDCap-based online survey was used to collect data [23].

### Measures

Descriptive characteristics included demographics such as age (years), sex (male/female), height (m), weight (kg), highest level of education (*in ascending order:* no school diploma, secondary school, apprenticeship, qualifying for university admission, or academic degree), and relationship status (single, in a relationship, married, divorced, or widowed). Participants were asked whether they engaged in bodybuilding, resistance training, and/or endurance (multiple choice questions). Participants were asked if they tracked calories. The rating was based on an ordinal scale ranging from no calorie tracking (0) to partial calorie tracking (1) to complete calorie tracking (2).

Binge eating was assessed using the diagnostic questions of the validated German version of the Eating Disorder Examination-Questionnaire [24, 25]. Participants were asked how many episodes of overeating occurred with concurrent loss of control in the last 28 days. The DSM-5 criteria considered it binge eating if participants had binge eating episodes on at least four of the previous 28 days.

The Muscle Dysmorphic Disorder Inventory (MDDI) was used to assess MD psychopathology [26]. This 13-item questionnaire contains three subscales: *drive for size* (5–25), *appearance intolerance* (4–20), and *functional impairment* (4–20). Total scores range from 13 to 65, with higher scores indicating more severe MD psychopathology. We used the validated German version [18], which showed a total score of Cronbach´s alpha of *α* = 0.75. In this study, Cronbach´s alpha was α = 0.70. A total score of > 39 was set as a cutoff to define an “MD at-risk” state for both men and women [27, 28].

The *Drive for Thinness* is a unifactorial 7-item subscale of the Eating Disorder Inventory [29, 30]. Total scores range from 7 to 42, with a higher score indicating a higher drive for thinness. Good internal consistency was observed in the current sample (α = 0.80).

The *Drive for Leanness Scale* [31] is a 6-item questionnaire with scores ranging from 5 to 30. Higher scores indicate a higher drive for leanness. Internal consistency yielded a Cronbach’s alpha of *α* = 0.77. As no German version was validated, the questionnaire was translated by the first author and then translated back to English by a native speaker. In the case of deviation from the original text, the German translation was discussed and adapted.

Good internal consistency was observed in the current sample (α = 0.81).

### Statistical methods

#### Descriptive analysis

Frequencies, means, and 95% confidence intervals were calculated for demographic data and all assessment instruments for both men and women.

#### Main analysis

To investigate the effect of MD psychopathology on binge eating, we calculated a hierarchical logistic regression analysis. Step 1 involved entering demographic data (sex, age, body mass index [BMI], education, and relationship status). In the second step, we entered sporting activity and proceeded with *calorie tracking*, the *Drive for Thinness Scale*, and the *Drive for Leanness Scale* in step 3. Step 4 involved entering *drive for size*, *appearance intolerance*, and *functional impairment*. Entering the three different subscales separately, rather than the total MDDI score, allowed for a more differentiated analysis of MD psychopathology.

Interactions between sex, *drive for thinness, drive for size, appearance intolerance*, and *functional impairment* were examined by simple slope analysis.

Average marginal effects (AME) were calculated to interpret the effect of the explanatory variables. AMEs reflect the average change of predicted probability for a one-unit change in continuous variables and for each observation in factorial variables while holding other covariates at their scores [32].

#### Secondary analysis

Predicted probabilities for binge eating were calculated for men and women at-risk of MD (MDDI > 39). Given the difference in MD psychopathology between men and women [18], predictions were calculated using local means. Comparisons of predicted probabilities were then performed using linear combinations of estimators [32].

#### Dropout analysis

Participants who did not complete all the questionnaires were excluded from further analysis. Before excluding them, group comparisons between participants with complete and incomplete data were performed using independent two-tailed welch-tests for continuous variables and two-sample tests of proportions for categorical data.

### Statistical software

Stata Statistical Software: Release 16.1. College Station, TX was used to analyze the data.

## Results

### Participants

Of the 7533 participants, 5905 (78.4%) were eligible and included in the study. The remaining participants (21.6%) did not complete all the questionnaires. Table 1 presents demographics, physical activity, and psychometric characteristics. Participants’ ages ranged from 18 to 72 years, and their BMIs from 13.3 to 66.4 kg/m^2^. Except for the MDDI, all assessment instruments recorded both minimum and maximum scores. The highest score of the MDDI was 63.

**Table 1:** Descriptive characteristics of the study sample

Variables	Men (n = 422)	Women (n = 5483)	Total (N = 5905)
*Demographics*			
Age, mean ± SD	26.1 ± 6.1	28.6 ± 7.1	28.4 ± 7.0
Body mass index, mean ± SD	26.2 ± 4.1	25.7 ± 5.9	25.8 ± 5.7
Marital status (%)			
Single	40.8	32.4	33.0
Relationship	48.6	47.3	47.4
Married	10	18.8	18.2
Divorced	0.7	1.5	1.4
Widowed	0	0.1	0.1
Education (%)			
No school diploma	0.2	0.1	0.1
Secondary school	5.5	2.4	2.6
Apprenticeship	26.3	21.0	21.4
Qualifying for university admission	27.5	33.8	33.4
Academic degree	40.5	42.6	42.5
*Sporting activity (%)*			
Resistance training	77.7	65.4	66.3
Bodybuilding	46.4	3.3	6.4
Endurance	37.0	54.9	53.6
*Psychometrics*			
Tracking calories (%)			
No	24.4	32.2	31.6
Yes	40.3	31.2	31.8
Partly	35.3	36.6	36.5
Drive for leanness (5–30), mean ± SD	21.8 ± 4.3	19.1 ± 5.1	19.3 ± 5.1
Drive for thinness (7–42), mean ± SD	19.1 ± 8.5	28.8 ± 8.8	28.1 ± 9.1
*MD psychopathology*			
MDDI total score (13–65), mean ± SD	34.8 ± 8.0	30.1 ± 6.6	30.4 ± 6.8
Drive for size (5–25), mean ± SD	14.4 ± 4.8	8.5 ± 3.4	8.9 ± 3.8
Appearance intolerance (4–20), mean ± SD	10.0 ± 3.8	12.9 ± 3.9	12.7 ± 3.9
Functional impairment (4–20), mean ± SD	10.4 ± 3.8	8.7 ± 3.7	8.8 ± 3.7
MD at-risk (%)	27.7	8.5	9.9
*Binge eating*			
Binge eating days (0–28), median (IQR)	1.0 (0.0, 3.0)	3.0 (0.0, 7.0)	3.0 (0.0, 6.0)
Binge eating DSM 5 (%)	24.9	45.1	43.7

Unless otherwise specified, mean and standard deviation are reported for continuous variables, and proportions are reported for categorical data

*MDDI* Muscle Dysmorphic Disorder Inventory, *MD* Muscle dysmorphia

### Dropout analysis

Participants excluded from data analysis owing to incomplete questionnaires were significantly older (*M* = 29, *SD* = 0.09) than the final study sample (*M* = 28.4, *SD* = 0.21; *t*(1702) = 2.95, *p* = 0.003). They were also significantly more often married (22%) than participants in the study sample (18%; *z* (3.28), *p* = 0.001) and reported significantly less calorie tracking (26.8%) than participants in the study sample (32%; *z* (− 2.87), *p* = 0.004). Reasons for early dropouts were not assessed.

### Main results

#### Hierarchical multiple logistic regression analysis

Table 2 summarizes the results from the final model of the hierarchical logistic regression analysis. Significant predictors of binge eating were age, being in a relationship or marriage, calorie tracking, the *drive for thinness*, and *appearance intolerance*. When adjusting *for drive for thinness* in step 2, no differences between men and women were found. BMI lost its significance when controlling for *appearance intolerance* in step 3.

**Table 2:** Results from the final model (step 3) of the hierarchical logistic regression on binge eating

Variable	OR [95% CI]	P
Sex: base male		
Female	0.92 [0.69, 1.25]	.6
Age	0.98 [0.97, 0.99]	< .001
Body mass index	1.01 [1.00, 1.02]	.12
Marital status: base Single		
Relationship	0.85 [0.74, 0.97]	.01
Married	0.76 [0.63, 0.91]	< .001
Divorced	0.92 [0.56, 1.52]	.75
Widowed	1.00 [empty]	.
Education: base No school diploma		
Secondary school	0.28 [0.05, 1.68]	.16
Apprenticeship	0.24 [0.04, 1.35]	.1
Qualifying for university admission	0.27 [0.05, 1.55]	.14
Academic degree	0.25 [0.04, 1.42]	.12
Resistance training: base No		
Yes	0.97 [0.85, 1.12]	.68
Bodybuilding: base No		
Yes	0.86 [0.64, 1.16]	.33
Endurance: base No		
Yes	1.05 [0.93, 1.19]	.39
Calorie tracking: base No		
Yes	0.66 [0.56, 0.77]	< .001
Partly	0.91 [0.78, 1.05]	.18
Drive for thinness	1.10 [1.09, 1.12]	< .001
Drive for leanness	1.01 [1.00, 1.02]	.17
Drive for size (MDDI)	1.00 [0.98, 1.02]	.87
Appearance intolerance (MDDI)	1.07 [1.05, 1.10]	< .001
Functional impairment (MDDI)	1.01 [0.99, 1.03]	.18
Constant	0.11 [0.02, 0.72]	.02
Observations	5895	
Total model	χ^2^ = 1382	< .001
Pseudo R^2^	0.171	

*MDDI* Muscle Dysmorphic Disorder Inventory

There were no significant interactions between sex, *drive for size, appearance intolerance, functional impairment,* and *drive for thinness*. There were no significant interactions between *drive for size, appearance intolerance,* and *functional impairment*. The interaction between *drive for thinness* and *appearance intolerance* was significant (p = 0.002) but yielded a negligible effect; thus, it was excluded from the final model.

#### AME

**Fig. 1:**
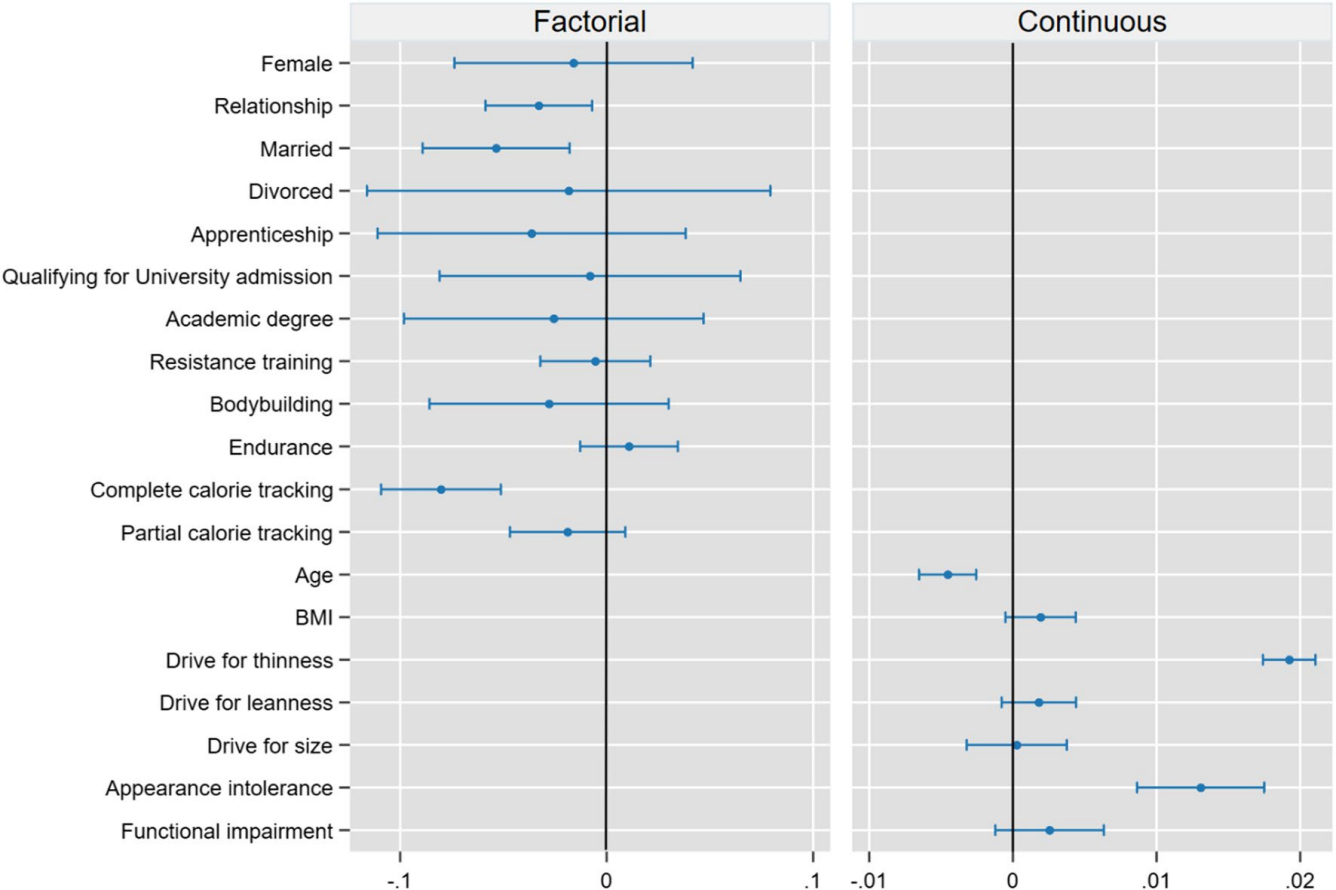
Average marginal effects of the explanatory variables. Whiskers represent the 95% CI. The line at zero indicates no average change in the predicted probability for a one-unit change in continuous variables and for each observation in factorial variables. In the interest of clear presentation, secondary school was chosen as the base for the variable education

Figure 1 displays the AME of the predictors.

Of the three subscales of the MDDI, only *appearance intolerance* was significant. On average, an increase in one unit of *appearance intolerance* was associated with a 1.3% (0.9–1.8%, p < 0.001) increase, and a full-range (4–20) increase was associated with a 21.9% (14.3–29.5%) increase in the predicted probability of binge eating. For *drive for thinness*, a one-unit change was associated with a 1.9% (1.7–2.1%, p < 0.001) increase in the probability of binge eating and a full-range (6–42) with a 64.8% (60.1–69.5%) increase. Age (one year) decreased the probability of binge eating by − 0.4% (− 0.6%, − 0.2%, p < 0.001), on average.

Compared to being single, being in a relationship (− 3.2%, CI − 5.8%, − 0.6%, p = 0.015) or married (− 5.3%, CI − 8.9%, − 1.8%, p = 0.003) was associated with a decreased probability of binge eating. There was no significant difference between being in a relationship or marriage. Calorie tracking was associated with a decreased probability of binge eating. The probability was reduced by − 8% (− 19.9%, − 5.1%) when calorie tracking was not used (p < 0.001). The probability was reduced by − 6.1% (− 8.8%, − 3.4%) when compared to partial calorie tracking (p < 0.001). There was no significant difference between “no calorie tracking” and “partial calorie tracking.”

#### MD psychopathology and predicted probabilities for binge eating in men and women

Although no differences were found between men and women when adjusting for covariates, it is unlikely that men and women at-risk of MD share the same average values for the predictors. As shown in Table 3, women at-risk of MD yielded significantly higher average scores on *drive for thinness* and *appearance intolerance*. Given the different characteristics of MD psychopathology, it is reasonable to assume that the predicted probability of binge eating associated with MD at-risk status differs between men and women.

**Table 3:** Comparison between men and women at-risk of MD

Variables	Men (n = 117)	Women (n = 465)	P
Age, mean ± SD	25.1 ± 5.3	26.4 ± 6.1	.025
BMI, mean ± SD	26.1 ± 4.0	24.2 ± 5.4	< .001
Marital status (%)			.24
Single	49.6	43.7	
Relationship	43.6	43.4	
Married	6.8	11.6	
Divorced	0.0	1.3	
Education (%)			.056
No school diploma	0.0	0.4	
Secondary school	10.3	4.1	
Apprenticeship	24.8	26.0	
Qualifying for university admission	26.5	34.2	
Academic degree	38.5	35.3	
Resistance training (%)	76.9	79.4	.56
Bodybuilding (%)	62.4	9.2	< .001
Endurance (%)	32.5	63.2	< .001
Tracking calories (%)			.086
No	13.7	22.6	
Yes	52.1	44.1	
Partly	34.2	33.3	
Drive for leanness, mean ± SD	23.9 ± 3.9	22.4 ± 4.7	.001
Drive for thinness, mean ± SD	19.8 ± 8.3	35.2 ± 6.8	< .001
MDDI total score, mean ± SD	44.7 ± 4.5	43.3 ± 3.3	< .001
Drive for size, mean ± SD	19.0 ± 3.5	13.1 ± 3.8	< .001
Appearance intolerance, mean ± SD	11.8 ± 3.1	16.1 ± 2.7	< .001
Functional impairment, mean ± SD	13.8 ± 3.3	14.1 ± 3.4	.45

Mean and standard deviation are reported for continuous variables and proportions are reported for categorical data. Comparisons between men and women were performed using the two-tailed independent sample t-test for continuous data and χ^2^ tests for categorical data. The means and proportions were multiplied by the corresponding coefficients of our model to calculate the probability of binge eating for men and women at-risk of MD

*BMI* body mass index, *MDDI* Muscle Dysmorphic Disorder Inventory

**Fig. 2:**
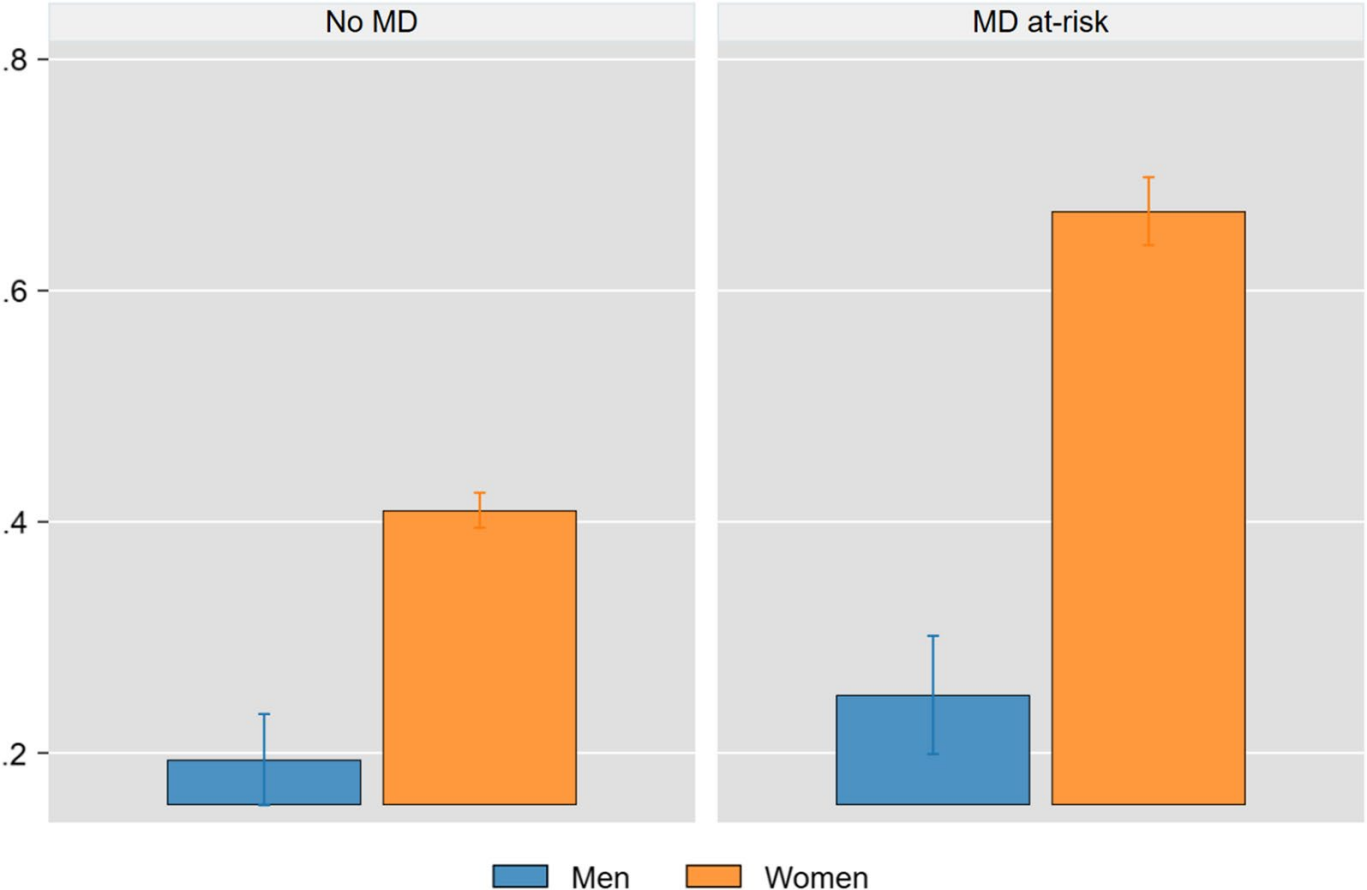
Predicted probabilities for men and women with and without MD at-risk. Predicted probabilities were calculated using local means. Significant differences are observed between men and women at-risk of muscle dysmorphia (MD) (Δ 41.9%, p < .001), between men without risk of MD and men at-risk of MD (Δ 5.6%, p < .001) and between women without risk of MD and women at-risk of MD (Δ 25.9%, p < .001). Whiskers represent the 95% CI

Figure 2 displays the predicted probabilities for binge eating in men and women with and without MD at-risk status.

The predicted probabilities for binge eating in men and women not at-risk for MD were 19.4% (15.5–23.4%) and 41% (39.5–42.5%), respectively. According to our model, MD at-risk status yielded a predicted probability for binge eating of 25% (19.9–30.1%) for men and 66.9% (63.9–69.8%) for women. The difference was significant (p < 0.001). *Drive for thinness* explained 28.3% (25.9–39.6%, p < 0.001) of the difference between men and women, while *appearance intolerance* explained 5.7% (3.7–7.6%, p < 0.001).

On average, MD at-risk status was associated with a 5.6% (2.8–8.4%, p < 0.001) increase in the predicted probability for binge eating in men and a 25.9% (22.9–28.9%, p < 0.001) increase among women. *Appearance intolerance* accounted for 3.4% (2.3–4.6%) of the difference in men and 4.5% (3–6.1%) in women. *Drive for thinness* accounted for 1.8% (1.6–1.9%, p < 0.001) of the difference in men and 13.6% (12.3–14.8%, p < 0.001) in women.

## Discussion

This study found that MD psychopathology was positively associated with binge eating in both men and women. Of the three subscales, however, only *appearance intolerance* yielded a significant effect, whereas *drive for size* and *functional impairment* did not. It is not merely the desire for a muscular body that is associated with binge eating, but rather the strong negative evaluation of one's body with the body-related feelings of shame and disgust. Although no differences were found between men and women when adjusting for covariates, MD at-risk status was associated with a greater increase in the predicted probability for binge eating in women than in men. Our original hypotheses were therefore supported by the findings.

Binge eating was significantly predicted by *drive for thinness* in both men and women, but not by *drive for leanness*. *Drive for leanness* refers to the control over a low body fat percentage and muscle visibility, whereas *drive for thinness* describes the control over low body weight. This conforms with Lang and Rancourt [33], who suggested that the drive for leanness is less maladaptive than the drive for thinness concerning the development of disordered eating. The interval between the lowest and the highest value was associated with a 64.8% (60.1–69.5%) increase in the probability of binge eating. An unexpected finding was that calorie tracking was significantly negatively associated with binge eating. This contradicts findings from Linardon and Messer [34], who found a positive association between the usage of calorie-tracking apps and objective binge eating in men. This result could be explained by the fact that our model was adjusted for *drive for thinness*. Without adjusting for *drive for thinness*, our analysis also showed a positive association between calorie tracking and binge eating. However, at the same *drive for thinness* values, calorie tracking seems to be negatively associated with binge eating. A possible explanation could be that calorie counting is mainly associated with restrained eating behaviors in sport and fitness oriented individuals when adjusted for *drive for thinness* [35]. Counting calories may also be avoided by individuals who suffer from binge eating, either because a binge eating episode cannot be accurately tracked or because knowing the exact number of calories consumed causes anxiety.

27.7% of men and 8.5% of women in our sample were at-risk for MD. According to our model, MD at-risk status yielded a predicted probability of binge eating of 25% for men and 66.9% for women. MD at-risk status resulted in a 5.6% increase in predicted probability for binge eating in men and a 25.9% increase in women. Although women at-risk for MD have lower MDDI scores than men, they are significantly more likely to engage in binge eating. There are two possible explanations for this. First, the clinical manifestation of MD at-risk status might differ depending on the characteristics of the three subscales. The probability of associated binge eating seems to be increased when self-depreciating thoughts and body-related feelings of shame and disgust predominate in the clinical presentation of MD. *Drive for size* was not significantly associated with binge eating; thus, it seems that it is not the desire for a muscular body itself that is associated with binge eating, but rather the subsequent negative evaluation of one's body. This is in line with longitudinal studies that demonstrated that body dissatisfaction is the most reliable predictor of disordered eating [36, 37]. For example, MD psychopathology between men and women seems to differ primarily in the *appearance intolerance* and *drive for size* subscales. Therefore, MD at-risk status is associated with different probabilities of binge eating in men and women.

The second reason might be the difference in drive for thinness between men and women. Not only did women have higher average values for *drive for thinness* than did men, but MD at-risk status was associated with a greater increase in *drive for thinness* in women than in men.

Concerning binge eating, *appearance intolerance* and *drive for thinness* have different relevance to men and women, as the increased probability of binge eating associated with MD at-risk status is mainly accounted for by *appearance intolerance* in men and *drive for thinness* in women.

Appearance intolerance is not specific to MD but is a key diagnostic feature for both MD and EDs. The more specific feature of MD, the *drive for size*, was not associated with binge eating. Thus, it seems that intentional, periodic overeating (“bulking”), as often practiced in the context of muscularity-oriented eating, is not associated with binge eating. In particular, the strong negative evaluation of one's body along with a high drive for thinness resulted in binge eating. Since a substantial proportion of men and women showed binge eating at an MDDI cutoff of > 39, it can be assumed that either the focus on dieting in MD can no longer be considered merely secondary [7], or that the associated binge eating corresponds to a comorbid ED. Since purging was not examined in this study, it cannot be determined whether the associated binge eating corresponds to uncompensated or compensated binge eating. The high proportion of binge eating in women at-risk of MD and the fact that exercise is a pivotal feature in the pursuit of the muscular ideal leads us to speculate that in women, MD at-risk closely resembles bulimia nervosa. To test this hypothesis and to capture the full spectrum of bulimic features associated with MD, future research should investigate possible purging behavior associated with MD.

Regardless of diagnostic classification, our findings support the assertion that bulimic features, particularly binge eating episodes, should form part of the assessment of MD [19]. As the drive for thinness and the pursuit of the muscular ideal are not mutually exclusive [12], it is also important to consider the fear of weight gain and the desire for a lean and thin figure when assessing possible pathologies in the pursuit of the muscular ideal.

Several limitations need to be considered. First, the validity of the diagnoses is not ensured, as no clinical interviews were conducted. Second, the MDDI cutoff > 39, although commonly used in studies [27, 28], has not been clinically validated; thus, it is unclear whether those with an MDDI > 39 meet the diagnostic criteria for MD. This holds true especially for women. Third, generalizability is limited because of the nonclinical, non-random sample, consisting of participants who followed fitness influencers on social media. Fourth, by using cross-sectional data, we are precluded from drawing causal conclusions from the associations we found. Fifth, those who did not meet the inclusion criteria differed significantly from the study sample concerning age, marital status, and calorie tracking. However, the difference was minimal. The differences in predicted probability for binge eating owing to age, marital status, and calorie tracking were − 0.003%, − 0.2%, and − 0.4%, respectively. Further studies are needed to replicate our findings using a prospective design and a clinical sample.

## Conclusion

The preliminary findings suggest that MD psychopathology is positively associated with binge eating in both men and women. MD at-risk status was significantly associated with an increased probability of binge eating. In men, it was mainly accounted for by appearance intolerance; in women, it was mainly accounted for by the concurrent increase in drive for thinness. Therefore, both MD psychopathology and drive for thinness should be considered when assessing disordered eating related to the muscular ideal. The assessment of bulimic features, particularly binge eating episodes, should form part of the assessment of MD.

## Data Availability

The datasets used and/or analyzed during the current study are available from the corresponding author on reasonable request.

## Abbreviations

AN: Anorexia nervosa
AME: Average marginal effects
BMI: Body mass index
DSM-5: Diagnostic and Statistical Manual of Mental disorders
ED: Eating disorders
MD: Muscle dysmorphia
MDDI: Muscle Dysmorphic Disorder Inventory
